# Corticosteroid-Induced Psychosis: A Report of Two Cases and Review of the Literature

**DOI:** 10.7759/cureus.81515

**Published:** 2025-03-31

**Authors:** Stefano Canessa-Muñoz, Silvia Yelmo-Cruz, Ana Hamilton-Lopez, Claudia Baez-Marrero

**Affiliations:** 1 Psychiatry, Hospital Universitario de Canarias, La Laguna, ESP

**Keywords:** affective, cognitive, corticosteroid, neuropsychiatric side effects, organic, psychosis

## Abstract

Glucocorticoids, prescribed for their anti-inflammatory and immunosuppressive effects, are linked to psychiatric side effects, including corticosteroid-induced psychosis. This condition may manifest as affective, organic, or cognitive disturbances, and although the exact pathophysiology is not fully understood, it is believed that corticosteroids impact the hippocampus and hypothalamic-pituitary-adrenal axis, causing neuropsychiatric changes. This report presents two cases of psychosis following corticosteroid use: a 59-year-old man with rheumatoid arthritis and a 62-year-old woman with asthmatic bronchitis. Both cases demonstrated rapid symptom improvement with antipsychotic treatment, emphasizing the need for early intervention. Despite the unclear pathophysiology, corticosteroid-induced psychosis generally has a positive prognosis when promptly managed with drug discontinuation and pharmacological treatment.

## Introduction

Glucocorticoids (such as prednisone, prednisolone, and methylprednisolone) are used worldwide for their anti-inflammatory and immunosuppressive properties, which are essential in managing numerous diseases [1]. Since Edward Kendall synthesized cortisone in 1940 and it began to be used for rheumatoid arthritis (RA), the side effects that this medication could produce became evident. The use of glucocorticoids in RA is to provide a rapid symptomatic relief by inhibiting inflammation. It also delays the onset and progression of radiographic joint damage [2]. Short-term use of oral glucocorticoids (within the first 30 days) is associated with an increased risk for sepsis, venous thromboembolism and fracture. Long-term use is associated with hypertension, bone fracture, cataract and gastrointestinal side effects [3,4]. It has been reported in different studies that psychiatric side effects are correlated with the use of high oral prednisone doses, usually higher than 40 mg/day. On the other hand, no correlation has been described between dose and diabetes or gastrointestinal side effects [5]. Psychiatric reactions can range from mild reactions, as an improved sense of well-being, anxiety or sleep disturbances, to severe conditions, like corticosteroid-induced psychosis.

The incidence of psychiatric complications varies widely: while 6% of cases involve severe reactions, up to 72% may experience symptoms if mild reactions are included such as insomnia, emotional lability or mood changes [6]. Psychiatric side effects can present as an affective form (75% of patients, predominantly depressive or manic), as an organic form (24% of patients, as psychosis) and as a cognitive form (1% of patients, as memory lapses or disorientation) [5]. Rome and Braceland defined four categories or degrees of psychiatric symptoms, with grade 1 presenting with a general feeling of well-being, increased energy and clarity of thought, up to grade 4, in which there are hallucinations, delusions and extreme variations in mood [7]. Although corticosteroid-induced psychosis is well-documented, it still lacks a fully defined pathophysiological model. It has been proposed that stress induced in the hypothalamic-pituitary-adrenal (HPA) axis by exogenous corticosteroid use affects the regulation of hormones like cortisol, negatively impacting the hippocampus and cognitive function. Additionally, increased dopamine production, possibly mediated by the induction of tyrosine hydroxylase, could contribute to the development of psychosis, as postulated in the stress-vulnerability model of schizophrenia [8].

## Case presentation

We report the cases of two patients who developed psychotic symptoms associated with corticosteroid use.

The first case is a 59-year-old Caucasian man who was admitted to the emergency department (ED) with agitation and psychotic behavior that had developed over 72 hours. He had RA diagnosed at age 45 and had discontinued treatment with methotrexate 10 mg/week orally, by his own decision, three years prior to admission. He had no personal or family psychiatric history nor history of substance abuse. Symptoms initiated with irritability, followed by persecutory ideas concerning his neighbours, as well as auditory and visual hallucinations (voices that threatened to kill him, and seeing shadows on the wall, respectively). These symptoms coincided with the abrupt discontinuation of corticosteroid treatment with prednisone (90 mg/day orally for three days), which had been prescribed for a RA flare-up with involvement of the metacarpophalangeal joints of both hands. Blood tests (blood count, biochemistry, coagulation, ethanol levels, thyroid hormones, serological tests for syphilis, HIV, hepatitis B virus (HBV) and hepatitis C virus (HCV)), urine drug screen (cannabis, cocaine, opiates, amphetamine, methamphetamine and methadone), brain CT scan, and brain MRI showed no significant abnormalities. Delirium was ruled out because the patient was alert, and he had well-structured and non-fluctuating symptoms. Risperidone 0.5 mg orally and diazepam 5 mg orally both at bedtime were started. After 48 hours in the ED, the patient was transferred to the Psychiatry unit, and risperidone was increased up to 3 mg orally at bedtime. He was discharged 11 days later, with complete resolution of psychotic symptoms. He was diagnosed with a corticosteroid-induced psychotic disorder. At admission, the Brief Psychiatric Rating Scale scored 75 points and at discharge, it was 3 points. Risperidone 3 mg orally at bedtime was prescribed at discharge treatment, with a referral for outpatient follow-up.

The second case concerns a 62-year-old Caucasian woman who was admitted to the ED with psychotic symptoms and behavioral changes, including delusional thoughts (believing she was very rich and the girlfriend of the president of the government), pressured speech, sexual comments toward men, insomnia and psychomotor agitation. She had no personal or family psychiatric history or substance abuse. One week prior to the onset of these symptoms, she had been prescribed prednisone 60 mg/day orally for three days due to asthmatic bronchitis followed by a gradual dose reduction: 30 mg/day for two days and then 15 mg/day orally for the last two days. She was also taking salbutamol (three inhalations every eight hours) and ipratropium bromide (three inhalations every eight hours). Blood tests - including blood count, biochemistry, coagulation, ethanol levels, thyroid hormones, serological tests for syphilis, HIV, HBV and HCV - along with brain CT scan were normal. Urine drug screen (cannabis, cocaine, opiates, amphetamine, methamphetamine and methadone) was negative. Delirium was ruled out as she was alert and her symptoms were non-fluctuating. A diagnosis of corticosteroid-induced manic episode with psychotic symptoms was made. Prednisone was discontinued and the patient started olanzapine 5 mg/day orally, with outpatient follow-up arranged. Young Mania Rating Scale (YMRS) was 31 points. However, one week later, the patient was brought by her son, who reported she had discontinued olanzapine and continued to exhibit manic symptoms (sexual comments and insomnia); YMRS was 30 points. She was admitted to the psychiatric ward, where treatment with olanzapine 5 mg/day orally was restarted, leading to rapid symptom improvement. She was discharged after 10 days with a YMRS of 5 points.

In both cases, a differential diagnosis was made to rule out primary psychotic or mood disorders, but the temporal criteria were not met, and a secondary cause - prednisone - was identified. Patient characteristics are summarized in Table 1.

**Table 1:** Clinical characteristics of patients

Characteristic	Patient 1	Patient 2
Age	59 years	62 years
Sex	Male	Female
Underlying Condition	Rheumatoid arthritis	Asthmatic bronchitis
Corticosteroid Dosage	Prednisone 90 mg/day (orally) for 3 days	Prednisone (orally) 60 mg/day for 3 days, then 30 mg/day for 2 days and then 15 mg/day for 2 days
Time to Symptom Onset	Six days after starting prednisone and three days after abrupt discontinuation	One week after starting prednisone
Symptoms	Agitation, irritability, persecutory delusions, auditory and visual hallucinations	Megalomaniacal ideas, pressured speech, psychomotor agitation, sexual comments, insomnia
Diagnosis	Corticosteroid-induced psychotic disorder	Corticosteroid-induced manic episode with psychotic symptoms
Treatment	Risperidone 3 mg/day (orally), diazepam 5 mg (orally)	Olanzapine 5 mg/day (orally)
Outcome	Full resolution in 11 days, outpatient follow-up	Resolution in 10 days, outpatient follow-up

## Discussion

The exact mechanism by which corticosteroid-induced psychosis occurs remains unclear. It has been proposed that corticosteroids affect the central nervous system, particularly the hippocampus and hypothalamus, causing alterations that affect thought, emotions, and behavior. The two cases presented highlight different forms of corticosteroid-induced psychosis, confirming the relationship between corticosteroid therapy and neuropsychiatric alterations [8].

In corticosteroid-induced psychosis, the hippocampus plays a crucial role in modulating the effects of glucocorticoids. While animal studies have shown dendritic atrophy and neuronal loss, postmortem studies in humans have not shown significant morphological changes in the hippocampus, although alterations in synaptic organization and reactive astrogliosis in the CA1 and CA3 areas of the hippocampus have been observed [9]. These functional changes in the hippocampus translate into cognitive dysfunctions, such as memory and attention problems [10], which are seen in patients treated with corticosteroids, as observed in both cases. The hippocampus also plays a key role in regulating the hypothalamic-pituitary-adrenal axis, and the administration of exogenous corticosteroids can suppress this axis, altering its regulatory function. Furthermore, hypothalamic involvement may be responsible for symptoms such as disturbances in sleep, food intake, body temperature, and emotional disturbances like irritability, aggression, and depression [11], symptoms that were observed in both cases. Corticosteroids affect the dopaminergic and serotonergic systems, with dopaminergic pathway hyperactivity being implicated in motivational deficits, anhedonia, and reward system dysfunction. A reduction in serotonin release contributes to manic, depressive, or mixed symptoms. Alterations in the cholinergic system have also been described, which could have implications for potential effects on cognitive functions [11].

The intensity and onset of neuropsychiatric symptoms induced by corticosteroids largely depend on the dose and duration of treatment. In the first case, the man with RA developed acute psychosis following an abrupt withdrawal of prednisone, suggesting that corticosteroid withdrawal can also be a trigger for psychosis [12]. He experienced auditory and visual hallucinations, as well as persecutory delusions, a classic presentation of corticosteroid-induced psychosis [13]. Treatment with risperidone was effective, with complete resolution of symptoms at discharge, highlighting the importance of early pharmacological intervention for recovery.

In the second case, the patient developed a manic episode after a short course of prednisone, underscoring the importance of both dose and duration of treatment. The rapid improvement of symptoms with olanzapine demonstrates that pharmacological intervention is essential [14]. Discontinuation of antipsychotic medication in this case was a key factor in symptom recurrence, emphasizing the need for close follow-up.

It is important to note that symptoms can appear rapidly after exposure, even with low doses or local administrations, such as epidural or intra-articular injections [15]. However, literature indicates a dose-response correlation, with doses above 40 mg/day of oral prednisone or equivalent being associated with a higher incidence of psychosis [16]. Interestingly, no consistent relationship has been observed with prior psychiatric history, but there is a higher incidence in women [16].

To diagnose substance- or medication-induced psychosis, such as corticosteroid-induced psychosis, psychotic symptoms must appear soon after medication exposure and cause significant functional impairment, according to Diagnostic and Statistical Manual of Mental Disorders, Fifth Edition (DSM-5) criteria [17].

The prognosis of corticosteroid-induced psychosis is generally favorable, with most patients showing remission of symptoms after discontinuation of corticosteroid treatment. In both cases presented, the patients showed improvement after receiving antipsychotics, such as risperidone and olanzapine, supporting the use of these drugs in the treatment of corticosteroid-induced psychosis.

The primary treatment for corticosteroid-induced psychosis is the gradual reduction of the corticosteroid dosage. When this is not possible, or when symptoms are severe, the administration of psychotropic medications is recommended. Effective treatments described in the literature include antipsychotics, mood stabilizers, and antidepressants. Both typical (haloperidol) and atypical (olanzapine, risperidone and quetiapine) antipsychotics have been shown to be effective. Among mood stabilizers, lithium carbonate, valproic acid, carbamazepine or lamotrigine can be used prophylactically in patients with a history of corticosteroid-induced psychosis who require further corticosteroid treatment. In treatment-resistant cases, combinations of mood stabilizers and antipsychotics, such as valproic acid or lithium with risperidone or olanzapine, have been used with good clinical outcomes [12,18].

Continuous psychiatric follow-up is necessary, especially in patients with psychiatric histories or those who discontinue antipsychotic treatment. Tricyclic antidepressants and antipsychotics with strong anticholinergic activity should be avoided due to their potential to cause confusion, blurred vision, constipation, dry mouth, dizziness or loss of balance [19]. Fortunately, the prognosis is generally favorable, with complete remission in over 90% of patients within six weeks of treatment [14].

At present, there is no clear way to predict which individuals will develop neuropsychiatric symptoms. However, blood-brain barrier damage and hypoalbuminemia are the most validated risk factors [20].

## Conclusions

Corticosteroid-induced psychosis is a relatively common disorder in general hospitals but is often underestimated in patients receiving high-dose (more than 40 mg/day of prednisone or equivalent) or long-term corticosteroid treatment. The two clinical cases highlight the diverse psychiatric manifestations of this condition, ranging from psychotic symptoms to manic episodes.

Early identification and proper treatment are crucial for a favorable prognosis, as timely discontinuation of corticosteroids and appropriate pharmacological management with antipsychotics can lead to full symptom resolution.

While the exact pathophysiology remains unclear, evidence suggests that corticosteroids disrupt the hypothalamic-pituitary-adrenal axis and hippocampal function, contributing to neuropsychiatric symptoms. Given the potential for symptom recurrence upon corticosteroid re-exposure or abrupt withdrawal, close monitoring and individualized treatment strategies - such as olanzapine, risperidone or lithium - are essential.

Further research is needed to refine risk stratification and optimize management approaches to prevent and mitigate corticosteroid-induced psychiatric complications.
